# Upper cervical chiropractic management of a patient with headaches, neck pain and a history of traumatic internal carotid artery compromise: a case report

**DOI:** 10.1080/10669817.2026.2684728

**Published:** 2026-06-15

**Authors:** Mychal Beebe, Ankur Tayal

**Affiliations:** a Private Practice, Portsmouth, NH, USA; b Sherman College of Chiropractic, Spartanburg, SC, USA

**Keywords:** Upper cervical, chiropractic, artery dissection, case report

## Abstract

**Objective:**

This case describes the upper cervical chiropractic management of a patient with neck pain and headaches with a history of cervical artery compromise following a motor vehicle accident.

**Background:**

A 54-year-old male with neck pain and headaches was seen in a chiropractic office. Eight months prior to his initial visit, the patient was involved in a motor vehicle accident (MVA) which resulted in partial dissection of his left internal carotid artery. The patient reported severe cervicalgia, headaches and painful restricted cervical motion. Physical exam and cervical imaging assessed acceptable risk for treatment and indicated altered axis of motion in the first cervical vertebrae (C1) relative to the occiput (Co).

**Outcomes:**

The patient was examined over a 6 month period and received a total of 5 Blair upper cervical chiropractic adjustments. Physical exam findings, the Numerical Rating Scale (NRS), the Neck Pain Disability Index (NDI) and the RAND-36 outcomes were used to track progress. The NRS, NDI and RAND-36 demonstrated positive changes in presenting symptoms.

**Conclusion:**

More investigation is needed to understand the relationship between a history of traumatic cervical artery compromise and the effectiveness and safety of upper cervical chiropractic care utilizing Blair protocol to manage latent symptoms.

## Background

This case reports upper cervical chiropractic [1] management of a patient with neck pain and headaches with a history of cervical artery compromise following a motor vehicle accident.

A discussion exists regarding the association of cervical spinal manipulative therapy (SMT) and cervical artery compromise. It has been reported that SMT is an independent risk factor for vertebral artery dissection [2]. In one study, chiropractic manipulation of the cervical spine was performed prior to the diagnosis of dissection in 20 patients: 5 had carotid artery dissection (CAD), 14 had vertebral artery dissection (VAD), and 1 had both [3]. Contrarily, Cassidy et al. observed 818 VAD victims matched with 3164 controls and found no evidence that seeing a chiropractor was linked with causality to the onset of vertebral artery compromise. It was found that no excess risk of vertebral artery compromise is associated with chiropractic care compared to the risk of primary care [4].

Carotid artery dissection following blunt cervical trauma is a rare injury occurring in 0.02 to 0.67% of cases [5]. The incidence of vertebral artery dissection is even less frequent than carotid with the true incidence unknown, as many cases are asymptomatic or mild at most [5,6]. Following a motor vehicle accident, neck pain and headache are the most frequently reported features and have a substantial impact on the onset of chronic neck pain and associated disability [7]. Neck pain, headaches and disability associated with motor vehicle accidents have high co-morbidity and cost to society [4,8]. Chiropractic care is reported to be a highly cost‑effective option for neck pain [9].

Upper cervical chiropractic care utilizing a specific framework is presented as treatment for this case once appropriate screening procedures of risk assessment utilizing verbal history, exam and prior imaging were performed and patient consent was obtained.

## Case description

A 54-year-old Caucasian male presented with primary complaints of neck pain and headaches which prevented him from working as a structural engineer in which climbing towers required extension of the cervical spine. He also reported discomfort when sitting in one place for 2 hours or more and difficulty in checking his blind spots while driving.

Eight months prior to seeking upper cervical chiropractic care, the patient was involved in a rear‑end motor vehicle crash at approximately 30 mph. On the day of the MVA, the patient went to the emergency room (ER) with a complaint of neck pain and headache. After an exam and cervical x-rays, the patient was discharged. Two days later, the patient began to notice left visual blurring, left facial numbness and facial droop, in addition to his previous symptoms. When the patient presented to the emergency department 6 days after the MVA, the ER report noted that the facial symptoms were present; however, no visual symptoms remained. All exam findings were normal except a decrease of light touch on the left face and left ptosis. A magnetic resonance angiogram (MRA) was ordered for the neck and head with and without contrast. The MRA report verified a left distal cervical internal carotid artery dissection extending approximately 1.5 cm from the second cervical vertebrae (C2) to the skull base with luminal narrowing approximately 40% at the skull base. Both vertebral arteries were normal. The MRI brain image was negative for acute stroke, and transcranial doppler was negative for emboli. Medication and stroke counseling were administered and follow up in 2 weeks recommended.

Four months after the MVA, the patient had a follow‑up MRI with and without contrast. The MRA reported the distal left internal carotid artery dissection near the skull base was no longer evident and that no significant stenosis was appreciated. During that exam the doctor assessed a complete review of systems including blood pressure, pulse, heart rate, physical exam with cervical palpation and neurologic exam. The patient continued to report neck pain and headaches, limiting his activities of daily living.

Eight months post MVA the patient reported to the chiropractic office with chronic constant moderate to severe cervicalgia, and chronic occasional severe headaches approximately twice per month. Relief was experienced from heat, ice, massage, lying down, and medications. Exacerbation noted with bending, coughing, lifting, movement of the head, sexual intercourse, and cervical extension and climbing which limited his work duties.

In October 2012, the International Federation of Orthopedic Manipulative Physical Therapists (IFOMPT) released a framework to provide guidance to clinicians for the assessment of the cervical region for potential of Cervical Arterial Dysfunction [10], which has since been revised as of 2020. The initial exam for this case occurred in 2013, and care was taken to address safety and consent with respect to current guidelines.

Upon examination, the patient appeared in moderate to mild discomfort but was alert and all signs of left facial weakness or ptosis resolved, visual assessment of cranial nerves appeared within normal. Palpation of the patient’s neck included his cervical spine and cervical vascular structures. Vascular structures were intact; however, muscle spasms of his right superior oblique muscle and right sternocleidomastoid muscle were appreciated. Cervical ranges of motion were restricted in bilateral passive lateral flexion and bilateral passive rotation with severe pain noted on passive extension and moderate pain on all lateral flexion and rotation. Cervical end range of motion including extension was not provocative of dizziness or vascular compromise, consistent with IFOMPT guidelines. Lumbar ranges of motion were restricted in active extension and active right lateral flexion with pain on active flexion and slight pain on active extension and bilateral active lateral flexion. Postural distortion noted with inferior left head tilt of ½ inch, right shoulder depression of 1 inch and right hip elevation of ½ inch and right head translation of 5/8 inch.

Per the IFOMPT cervical framework, a detailed health history was taken, cranial nerve, balance, gait and coordination integrity were assessed, cervical artery palpation was performed, and cervical end range of motion was evaluated, with a follow-up discussion of recommended procedures and risks. Given the clinical history of vascular pathology, it was critical to note that the follow‑up MRA reported resolution of the dissection as well as a health history and physical exam that removed the likelihood of current vascular compromise prior to moving forward with chiropractic management of the cervical spine.

The Blair Upper Cervical Procedure [11,12] was considered appropriate for this case, as the application of the manual adjustments to the cervical spine is non-manipulative, the patient remains in a neutral position, and the force profiles are a light quick toggle recoil applied to the cervical vertebrae. The toggle recoil adjustment utilizes a drop head piece with a total excursion approximately ¼ inch that, coupled with the neutral head piece, was hypothesized to minimize any deformation of cervical structures including vasculature and limit risk. The adjustment was delivered with the patient in a side laying position on a chiropractic toggle table. The posterior superior aspect of the left transverse process was contacted with the practitioners left pisiform. The practioner delivered a lateral to medial superior to inferior torqued toggle thrust along the individual slope angle of the right occipital condyle as measured from the right protracto radiograph.

The Blair Upper Cervical Procedure also encompasses tools for evaluation of the upper cervical spine. It was utilized to determine clinical indication of altered axis of motion in the cranio-cervical junction contributing to elevated pain perception, and altered cervical ranges of motion potentially contributing to headaches and altered sensory integration for the patient [13,14]. An altered axis of motion can be defined as a measurable deviation in the normal instantaneous center or plane of joint rotation within a spinal motion segment, resulting from changes in joint mechanics and/or neuromuscular control, and is often associated with abnormal afferent input from spinal tissues. Altered axis of movement of a vertebral segment coupled with changes in neurophysiologic function has been shown to have deleterious effects on cervical spine function and sensorimotor integration [15–17]. With this knowledge, application of the upper cervical chiropractic protocols was determined to be appropriate for the patient’s history of cervical artery compromise and latent symptoms.

The Blair procedure consists of paraspinal surface thermography, leg length inequality tests, also known as Prill tests [11], cervical syndrome [18] and Blair upper cervical specific radiographs [11]. During the initial exam, the patient demonstrated a prone leg length inequality measurement of right ½ inch, a positive left cervical syndrome and a positive right vertical Prill leg test.

**Figure 1.**
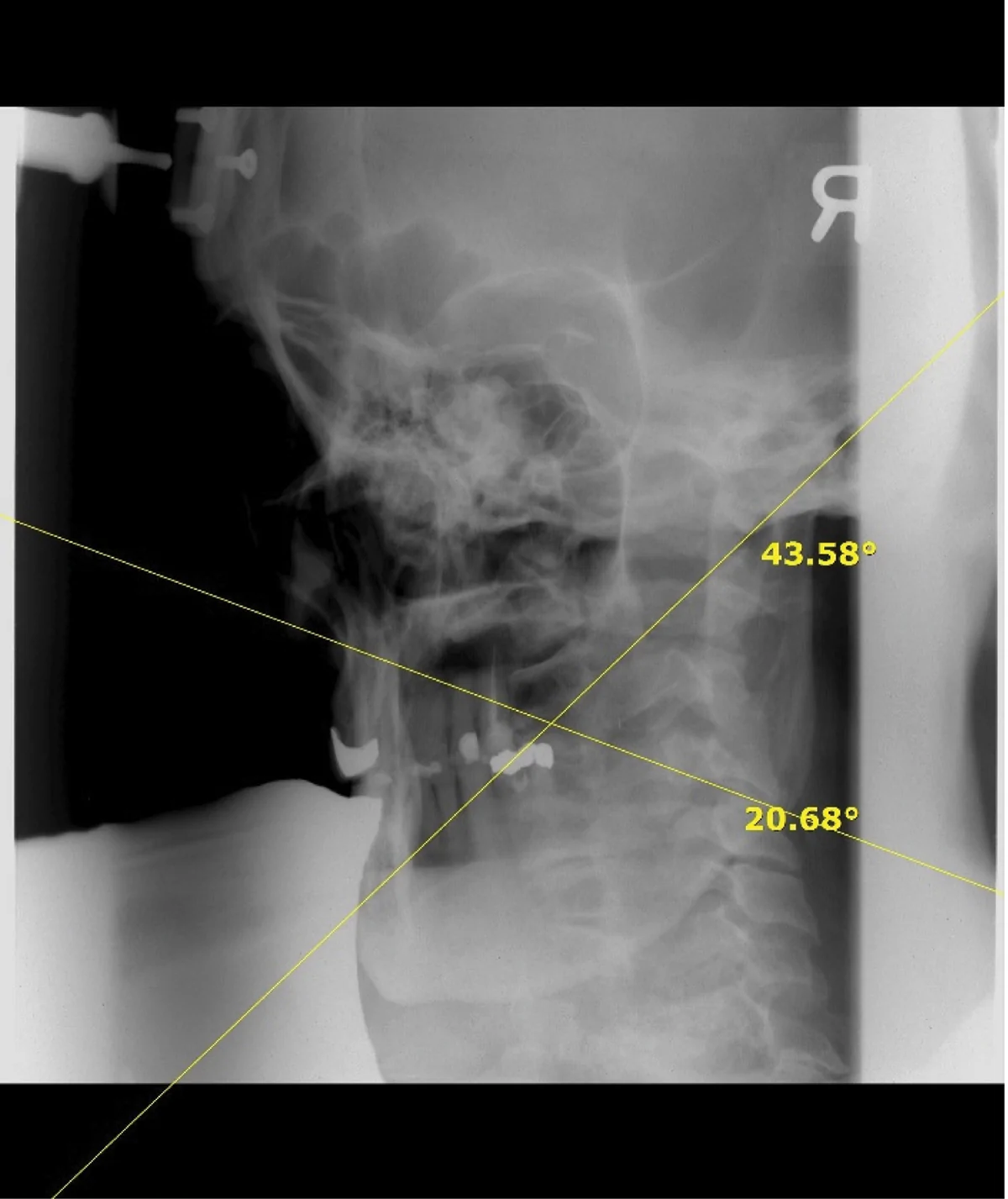
Left protracto view radiograph.

**Figure 2.**
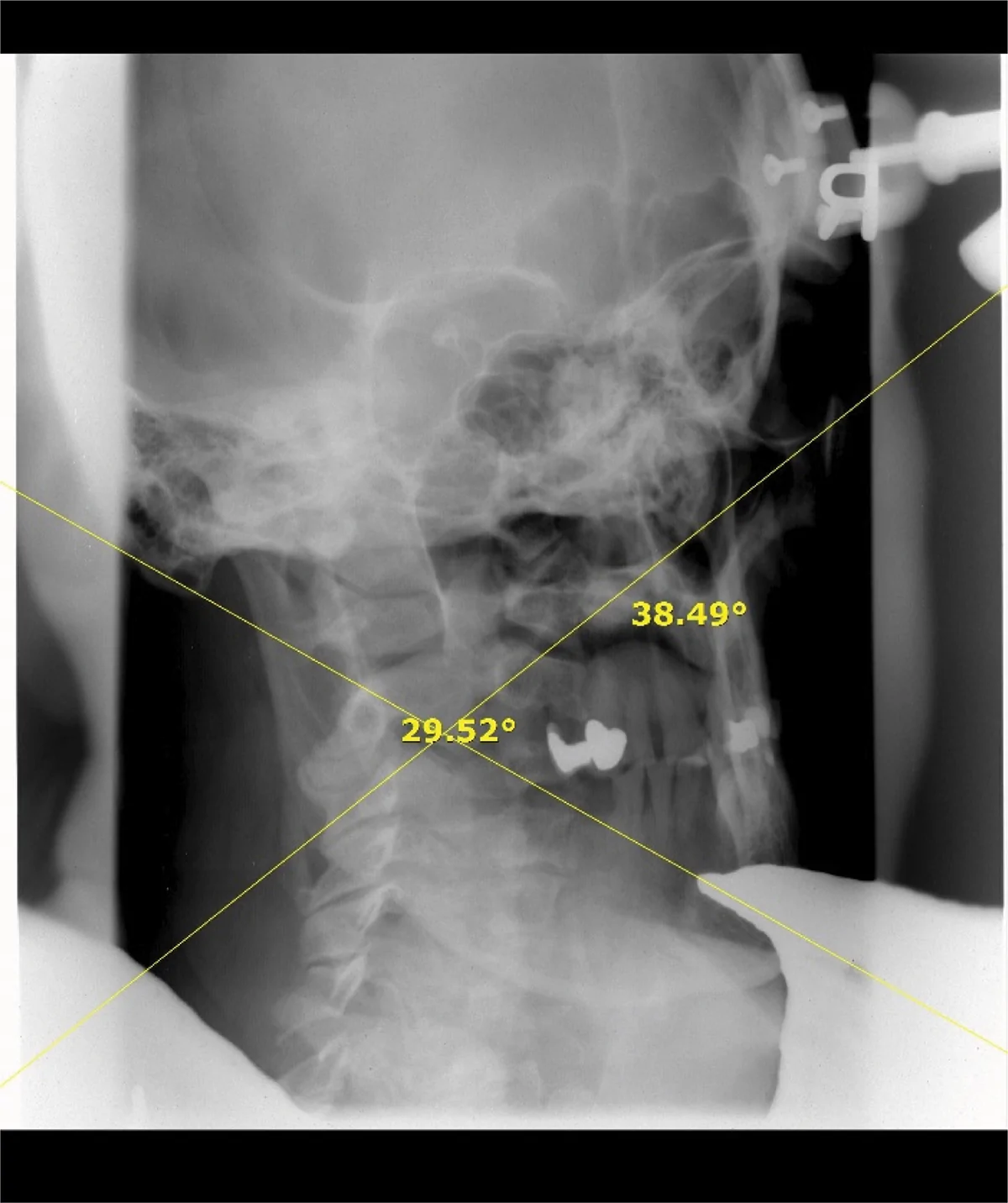
Right protracto view radiograph.

Upper cervical specific x-rays were taken including; Base posterior, A-P open mouth, lateral stereos, and bilateral protractos (Figures 1 and 2) as described by Blair upper cervical chiropractic procedures [11,15]. On the left protracto view, an overlap of the anterolateral margin of the left lateral mass under the left occipital condyle was demonstrated, which is denoted as an anterior superior left (ASL) C1 listing as measured relative to the skull. The x-rays help determine the altered axis of motion and the appropriate joint angles to apply the toggle recoil thrust. These tests are technique‑specific interpretations which helped frame the treatment choices for this patient.

X-Rays were also assessed for alignment and pathology of the bone, cartilage, and soft tissue structures of the cervical spine. Images were assessed for any contraindications for adjusting, as well as any indicators to modify the approach for any safety concerns.

Benefit and necessity of radiography are important to consider and contrast with safety and risk of ionizing radiation when manual adjustments are applied. In this case, the patient’s history of whiplash trauma and cervical artery dissection presented evidence and clinical rationale for necessary radiographs required to rule out contraindications and rule in safety to adjust [19]. Despite MRI and MRA already being performed, radiographs have been shown to be safe and necessary in not only ruling out pathology and contraindication but providing relevant information in manual spinal therapy. Considerations of excessive or harmful radiation were ruled out due to the benefits associated with the measurements obtained from the Blair series analysis [20,21].

On average, upper cervical chiropractic care has been reported to require fewer overall adjustments to the cervical spine compared to high‑velocity, low‑amplitude manipulation requiring extension, lateral flexion and rotation of the neck to achieve the same or better symptomatic outcomes [22].

## Outcomes

**Figure 3.**
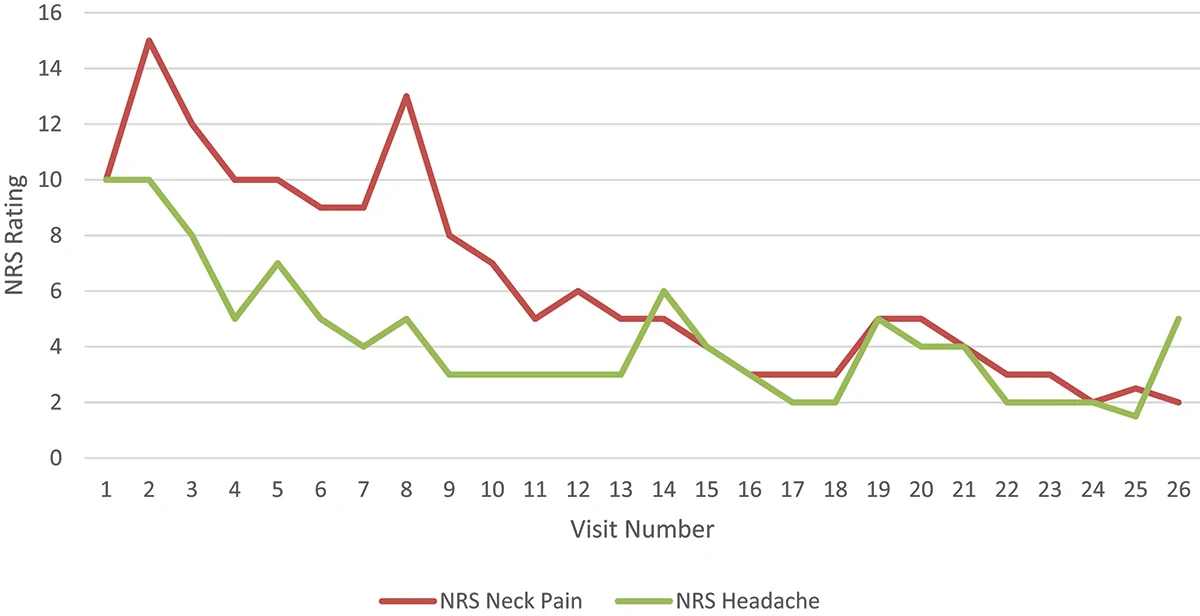
Patient reporting on neck pain and headache by visit using the Numerical pain rating scale (NRS).

Following the initial evaluation, the patient returned for treatment. Each office visit consisted of patient reporting on the 11-point Numerical Pain Rating scale [23], where the neck pain rating came down by 8 points and the headache rating came down by 5 (Figure 3). Additionally digital paraspinal thermography from T1 to the occipital shelf using a Tytron C-3000 scanner [24,25], prone leg length measurements were taken, and a cervical syndrome test was performed. If the patient demonstrated the characteristic consistent clinical findings indicating that the C1 vertebra altered axis of motion, a Blair Upper Cervical Toggle Torque adjustment was administered to the C1 vertebra.

The patient returned for a total of 26 visits over 6 months for follow‑up care. The frequency of visits began with 3 visits per week for 2 weeks, 2 visits per week for 4 weeks, 1 visit per week for 6 weeks and 2 visits per month for 3 months. On each office visits, the described protocol of ranges of motion, palpation, thermal scanning, and leg length inequality was performed. If indicated, a Blair upper cervical adjustment to the C1 vertebra was performed. If the cervical spine was deemed stable and presenting no altered axis of motion, no treatment was rendered.

The Blair upper cervical adjustment was delivered a total of 5 times, on visits 1, 5, 8, 12 and 19.

**Figure 4.**
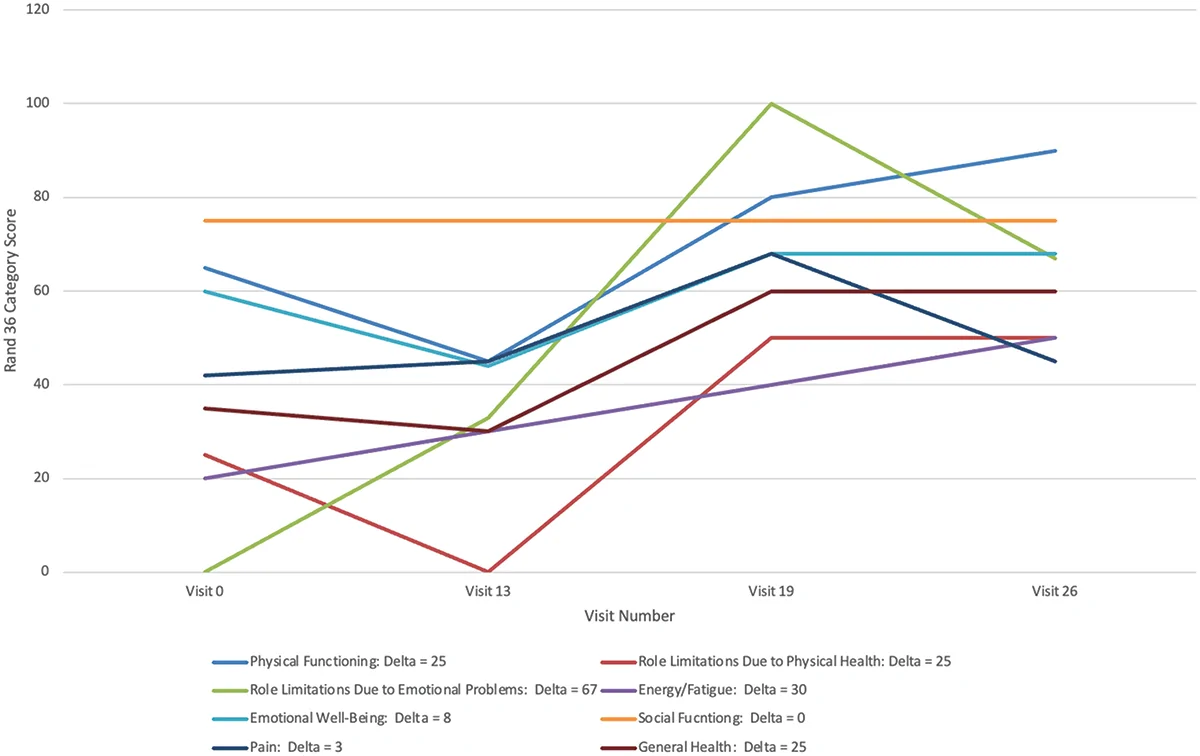
Patient reported quality of life outcomes over 26 visits using the Rand-36 survey.

**Figure 5.**
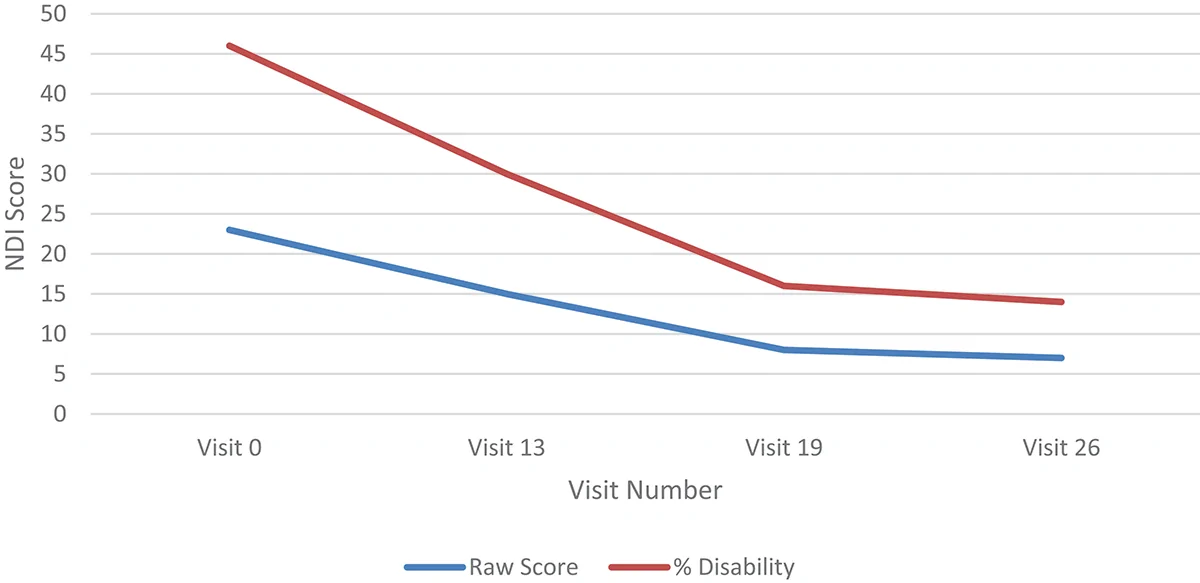
Patient reported neck pain disability (NDI) results as raw score and disability percentage.

A reevaluation exam was performed at visit 13, visit 19, and visit 26. Each reevaluation consisted of cervical manual palpation, cervical passive range of motion, active lumbar range of motion, postural assessment, paraspinal thermography, prone leg length test, cervical syndrome test and Prill test. The Rand-36 [26] was administered to track quality of life outcome measures (Figure 4), and the Neck Pain Disability Index [27] (NDI) was used track functional outcome. (Figure 5) Rand scores improved by 183 points over the 8 categories it measures, with the greatest improvement shown in role limitations due to emotional problems, which showed a 67 point improvement. Neck pain disability scores improved by 16 points, starting at a score of 23 which reduced to a 7, the disability reducing by 32%.

According to Pool et al., the minimal clinically significant change for the NDI is 10.5 points on the 50-point scale, and the minimal clinically significant change for the NRS is 4.3 points [28]. With respect to the NDI, this case demonstrated an overall change of 16 points, while the NRS revealed changes of 7.5 on neck pain and 8.5 on headache. Regarding the Rand-36 medical outcome study, all scores are 0–100 where a high score defines a more favorable health state [26].

## Adverse events

During the initial evaluation, the patient had been informed of all potential risks of the application of the upper cervical chiropractic treatment to the cervical vertebra. Reported adverse event occurred after the initial Blair upper cervical adjustment on visit 1 where the patient reported a 20–50% increase in neck pain over a 7-day period. After 7 days, the neck pain returned to baseline and then began to decrease. After the 7th visit Blair upper cervical adjustment, the patient reported a 30% increase in neck pain that lasted for 3 days. This reporting is consistent with the common occurrence of Delayed Onset Muscle Soreness (DOMS) and tissue regeneration associated with chiropractic care, particularly after whiplash injuries [29,30]. Subsequent interventions did not produce any further adverse events. These two adverse events over the 6-month treatment were limited to neck pain, and care was taken with examination to insure no concurrent vascular or neurologic sequala. As these events had a discreet timeline and the total trend of both subjective and objective outcomes was progressing, the care was considered to be appropriate.

## Discussion

In this case, upper cervical chiropractic utilizing the Blair protocol was an apparently safe and effective management tool for a patient with neck pain and headaches and a history of traumatic carotid artery dissection following a motor vehicle accident. An investigation of the current scholarly literature did not return any reports of upper cervical chiropractic management of a patient with a history of vascular compromise. However, there exist abundant references of cervical manipulation and mobilization of neck pain and headache [31].

The Blair Upper Cervical Protocol lacks the rigorous studies that would indicate clear causality between treatment and outcomes. Thus, these protocols and measurements were used in a clinical setting as a framework shape case management.

Neck pain and headaches are the two most common complications of motor vehicle accidents [7] and a natural progression of resolution cannot be discounted, due to time, placebo effect. Significant confounding factors include the natural history of neck pain and headache resolution; yet Gore et al. followed 205 patients for 10 years and found that 32% had moderate to severe residual pain. Patients who had a history of injury and had initially reported severe pain were the most likely to suffer [32].

Major weaknesses of this report include the uncontrolled external environment inherent in clinical practice, the lack of comparative control as well as possible unreported changes in work or home environment, and unreported concurrent treatment modalities. Additionally, outside of the IFOMPT cervical framework, blood pressure was not taken during the course of care.

## Conclusion

More investigation is needed to understand the relationship between a history of traumatic cervical artery compromise and the use of upper cervical chiropractic SMT Blair protocol to manage latent symptoms. This case does not demonstrate safety and efficacy for broad populations rather reports on an individual case who had a relatively recent history of vascular injury. Further study would need to be done to evaluate if the results are applicable to a wider population of neck pain and headache sufferers with a history of traumatic vascular compromise.

## References

[1] Woodfield CH, York C, Rochester, R.P., et al. Craniocervical chiropractic procedures – a précis of upper cervical chiropractic. J Can Chiropr Assoc. 2015;59(2):173–192. PMC4486989 26136610

[2] Smith WS, Johnston SC, et al. Spinal manipulative therapy is an independent risk factor for vertebral artery dissection. Neurology. 2003;60(9):1424–1428. doi: 10.1212/01.WNL.0000063305.61050.E6 12743225

[3] Dziewas R, Carsten K, Dräger B, et al. Cervical artery dissection- clinical features, risk factors, therapy and outcome in 126 patients. J Neurol. 2003;250(10):1179–1184. doi: 10.1007/s00415-003-0174-5 14586598

[4] Cassidy JD, Boyle E, Côté P, et al. Risk of vertebrobasilar stroke and chiropractic care. Spine (Phila Pa 1976). 2008;33(Supplement):S176–S183. doi: 10.1097/BRS.0b013e3181644600 18204390

[5] Rogers FB, Baker EF, Osler TM, et al. Computed tomographic angiography as a screening modality for blunt cervical arterial injuries: preliminary results. J Trauma. 1999;46(3):46;380–385. doi: 10.1097/00005373-199903000-00005 10088837

[6] Savitz SI, Caplan LR. Vertebrobasilar disease. N Engl J Med. 2005;352(25):2618–2626. doi: 10.1056/NEJMra041544 15972868

[7] Bunketorp L, Stener-Victorin E, Carlsson J. Neck pain and disability following motor vehicle accidents- a cohort study. Eur Spine J. 2005;14(1):84–89. doi: 10.1007/s00586-004-0766-5 PMC3476670 15241671

[8] Cote P, Cassidy JD. Is a lifetime history of neck injury in a traffic collision associated with prevalent neck pain, headache and depressive symptomatology? Accident Anal Prev. 2000;32():151–159. doi: 10.1016/S0001-4575(99)00117-7 10688471

[9] Niteesh C, Milstein A. Do chiropractic physician services for treatment of low back and neck pain improve the value of health benefit plans? An evidence-based assessment of incremental impact on population health and total care spending. Prepared for the Foundation for Chiropractic Progress. Mercer Health and Benefits LLC; 2009.

[10] Rushton A, Rivett D, Carlesso L, et al. International framework for examination of the cervical region for potential of cervical arterial dysfunction prior to orthopaedic manual therapy intervention. Man Ther. 2014;19(3):222–228. doi: 10.1016/j.math.2013.11.005 Epub 2013 Nov 23. PMID: 24378471. 24378471

[11] Susan H, Allison M. Upper cervical care in a nine-year-old female with occipital lobe epilepsy: a case study. J Up Cerv Chiropr Res. 2011;3:10–17.

[12] Muncy W, Forest T, Hubbard T, et al. Blair upper cervical specific technique. 2nd ed. Blair Upper Cervical Chiropractic Society; 2005.

[13] Owens E. Chiropractic subluxation assessment: what the research tells us. J Can Chiro Assoc. 2002;46(4):215–220.

[14] Henderson CN. The basis for spinal manipulation: chiropractic perspective of indications and theory. J Electromyogr Kinesiol. 2012;22(5):632–642. 10.1016/j.jelekin.2012.03.008 22513367

[15] Ambalavanar U, Wise R, Haavik H, et al. Selective improvement in neck and limb motor control outcomes following treatment of the upper neck and spine: a repeated measures cohort study. J Integr Neurosci. 2025;24(7):39548. doi: 10.31083/JIN39548 PMID: 40767017. 40767017

[16] Niazi IK, Navid MS, Merkle C, et al. A randomized controlled trial comparing different sites of high-velocity low amplitude thrust on sensorimotor integration parameters. Sci Rep. 2024;14(1):1159. doi: 10.1038/s41598-024-51201-9 PMID: 38216596; PMCID: PMC10786886. PMC10786886 38216596

[17] Taylor HH, Murphy B. Altered central integration of dual somatosensory input after cervical spine manipulation. J Manipulative Physiol Ther. 2010;33(3):178–188. doi: 10.1016/j.jmpt.2010.01.005 PMID: 20350670. 20350670

[18] Jackson R. The classic: the cervical syndrome. Clin Orthop Relat Res. 2010;468(7):1739–1745. doi: 10.1007/s11999-010-1278-8 PMC2881998 20177837

[19] Lopes MA, Coleman RR, Cremata EJ. Radiography and clinical decision-making in chiropractic. Dose Response. 2021;19(4). doi: 10.1177/15593258211044844 PMID: 34675758; PMCID: PMC8524714. PMC8524714 34675758

[20] Kent C. An evidence-informed approach to spinal radiography in vertebral subluxation centered chiropractic practice. Ann Vertebral Subluxation Res. 2017;2017(Aug 31):142–146.

[21] Arnone PA, McCanse AE, Farmen DS, et al. Plain radiography: a unique component of spinal assessment and predictive health. Healthcare (Basel). 2024;12(6):633. doi: 10.3390/healthcare12060633 PMID: 38540597; PMCID: PMC10970708. PMC10970708 38540597

[22] Eriksen K, Rochester RP, Hurwitz EL. Symptomatic reactions, clinical outcomes and patient satisfaction associated with upper cervical chiropractic care: a prospective, multicenter, cohort study. BMC Musculoskelet Disord. 2011;12():219. doi: 10.1186/1471-2474-12-219 PMID: 21974915; PMCID: PMC3204272. PMC3204272 21974915

[23] Farrar JT, Young JP Jr, LaMoreaux L, et al. Clinical importance of changes in chronic pain intensity measured on an 11-point numerical pain rating scale. Pain. 2001;94(2):149–158. doi: 10.1016/S0304-3959(01)00349-9 PMID: 11690728. 11690728

[24] Owens EF Jr Jr, Hart JF, Donofrio JJ, et al. Paraspinal skin temperature patterns: an interexaminer and intraexaminer reliability study. J Manipulative Physiol Ther. 2004;27(3):155–159. doi: 10.1016/j.jmpt.2003.12.019 PMID: 15129197. 15129197

[25] Hart J, Omolo B, Boone WR, et al. Reliability of three methods of computer-aided thermal pattern analysis. J Can Chiropr Assoc. 2007;51(3):175–185. PMID: 17885680; PMCID: PMC1978449. PMC1978449 17885680

[26] Hays RD, Morales LS. The RAND-36 measure of health-related quality of life. Ann Med. 2001;33(5):350–357. doi: 10.3109/07853890109002089 11491194

[27] Vernon H, Mior S. The neck disability index: a study of reliability and validity. J Manipulative Physiol Ther. 1991;14(7):409–415. 1834753

[28] Pool JJ, Ostelo RW, Hoving JL, et al. Minimal clinically important change of the neck disability index and the numerical rating scale for patients with neck pain. Spine (Phila Pa 1976). 2007;32(26):3047–3051. doi: 10.1097/BRS.0b013e31815cf75b PMID: 18091500. 18091500

[29] Senstad O, Leboeuf-Yde C, Borchgrevink C. Frequency and characteristics of side effects of spinal manipulative therapy. Spine (Phila Pa 1976). 1997;22(4):435–440; discussion 440–1. doi: 10.1097/00007632-199702150-00017 PMID: 9055373. 9055373

[30] Freeman MD, Croft AC, Rossignol AM. “Whiplash associated disorders: redefining whiplash and its management” by the Quebec task force. A critical evaluation. Spine (Phila Pa 1976). 1998;23(9):1043–1049. doi: 10.1097/00007632-199805010-00015 PMID: 9589544. 9589544

[31] Gross AR, , et al. A Cochrane review of manipulation and mobilization for mechanical neck disorders. Spine (Phila Pa 1976). 2004;29(14):1541–1548. doi: 10.1097/01.BRS.0000131218.35875.ED 15247576

[32] Gore DR, Sepic SB, Gardner GM, et al. Neck pain: a long-term follow-up of 205 patients. Spine (Phila Pa 1976). 1987;12(1):1–5. doi: 10.1097/00007632-198701000-00001 PMID: 3576350. 3576350

